# Modelling and analysis of influenza A (H1N1) on networks

**DOI:** 10.1186/1471-2458-11-S1-S9

**Published:** 2011-02-25

**Authors:** Zhen Jin, Juping Zhang, Li-Peng Song, Gui-Quan Sun, Jianli Kan, Huaiping Zhu

**Affiliations:** 1 Department of Mathematics, North University of China, Taiyuan 030051, China; 2 Chinese Center for Disease Control and Prevention, Beijing 10050, China; 3 Department of Mathematics and Statistics, York University, 4700 Keele Street, Toronto, Canada, M3J 1P3

## Abstract

**Background:**

In April 2009, a new strain of H1N1 influenza virus, referred to as pandemic influenza A (H1N1) was first detected in humans in the United States, followed by an outbreak in the state of Veracruz, Mexico. Soon afterwards, this new virus kept spreading worldwide resulting in a global outbreak. In China, the second Circular of the Ministry of Health pointed out that as of December 31, 2009, the country’s 31 provinces had reported 120,000 confirmed cases of H1N1.

**Methods:**

We formulate an epidemic model of influenza A based on networks. We calculate the basic reproduction number and study the effects of various immunization schemes. The final size relation is derived for the network epidemic model. The model parameters are estimated via least-squares fitting of the model solution to the observed data in China.

**Results:**

For the network model, we prove that the disease-free equilibrium is globally asymptotically stable when the basic reproduction is less than one. The final size will depend on the vaccination starting time, *T*, the number of infective cases at time *T* and immunization schemes to follow. Our theoretical results are confirmed by numerical simulations. Using the parameter estimates based on the observation data of the cumulative number of hospital notifications, we estimate the basic reproduction number *R*_0_ to be 1.6809 in China.

**Conclusions:**

Network modelling supplies a useful tool for studying the transmission of H1N1 in China, capturing the main features of the spread of H1N1. While a uniform, mass-immunization strategy helps control the prevalence, a targeted immunization strategy focusing on specific groups with given connectivity may better control the endemic.

## Introduction

**Figure 1:**
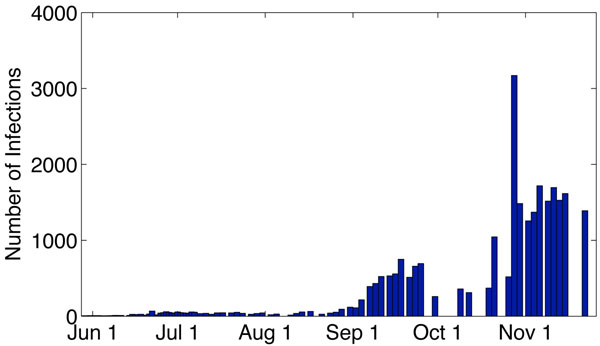
**The data of the Influenza A infection in China**. The data of the influenza A infection in China reported by Chinese CDC from June 1 to November 22, 2009.

In April 2009, a new strain of H1N1 influenza virus, referred to as influenza A (H1N1), was first detected in humans in the United States followed immediately by an outbreak in the state of Veracruz, Mexico. Since then, this new virus has kept spreading worldwide causing a global outbreak. As of December 20, 2009, it was reported by WHO that more than 208 countries and territories experienced the pandemic resulting in at least 11,516 deaths [1]. In China, the Circular of the Ministry of Health of the People’s Republic of China pointed out that as of December 31, 2009, the 31 provinces had reported 120,498 confirmed cases of H1N1 [2] . Of these confirmed cases, 118,244 had recovered, while 648 died. However, actual number of cases of people infected with the new virus is likely to be much higher than these numbers suggest, as most cases are not tested. Figure 1 shows the number of reported cases of H1N1 in China since June 2009. Similar to other influenza viruses, pandemic H1N1 typically results from person-to-person transmission through respiratory droplets generated by coughing and sneezing [3]. Symptoms usually last 4-6 days [4]. The infectious period for a confirmed case is defined as 1 day prior to the onset of symptoms to 7 days after onset. For a more detailed description of H1N1, see the Center for Disease Control and Prevention (CDC) [3], World Health Organization (WHO) [1], and Medscape’s H1N1 Influenza A (Swine Flu) Alert Center [4].

The H1N1 pandemic calls for action, and various mathematical models have been constructed to study the spread and control of H1N1. Fraser *et al.* estimated the basic reproduction number *R*_0_[5] in the range of 1.4 to 1.6 by analyzing the outbreak in Mexico, and earlier data of the global spread [6]. Nishiura *et al.* also estimated the reproduction number *R*_0_ but in the range of 2.0 to 2.6 for Japan [7]; they also estimated the reproduction number as 1.96 for New Zealand [8]. Vittoria Colizza *et al.* used a global epidemic and mobility model to obtain the estimation of the size of the epidemic in Mexico as well as that of imported cases at the end of April, 2009 [9]. Marc Baguelin *et al.* presents a real-time assessment of the effectiveness and cost-effectiveness of alternative influenza A (H1N1) vaccination strategies by a dynamic model [10]. H1N1, like many other infectious diseases, is intrinsically related to human social networks; it exhibits great heterogeneity in terms of the numbers and the pattern of contacts. The usual compartmental modelling in epidemiology generally assumes that population groups are fully and homogeneously mixed, but this does not reflect the real situation of the variation in the process of contact transmission. The epidemic modelling on complex networks has been attracting great interest, and various epidemic models on complex networks have been extensively investigated in recent years [11-17].

## The network model and parameters

**Figure 2:**
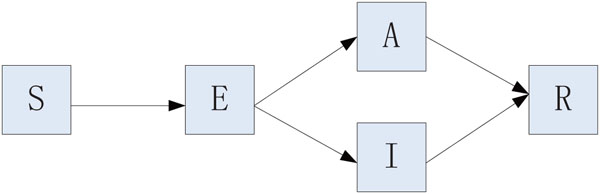
**The model.** Flow diagram of the transmission. Individuals may be Susceptible, Exposed, Asymptomatic, Infected or Recovered.

Based on the spreading process of H1N1, we propose an SEIAR model by classifying the population as susceptible (*S*), exposed (*E*), asymptomatically infected (*A*), symptomatically infected (*I*) and removed/immune (*R*)*.* The asymptomatically infected compartment contains those who fail to show noticeable symptoms or with light flu-like symptoms; they are not identified as H1N1 cases, but are able to spread the infection. We assume that a susceptible individual becomes infected if they come into contact with an asymptomatically or symptomatically infective individual. Then, the susceptible enters the exposed class *E* of those in the latent period. The period of incubation for H1N1 is 1-3 days [3]. After the latent period, the individual enters the class *I* or *A* of infectives, who are infectious in the sense that they are capable of transmitting the infection. When the infectious period ends, the individual enters the recovered class *R.* We assume that a removed individual will never become susceptible or infected again. In our model, new births, natural deaths and migrations are ignored. The flow diagram of the individuals is depicted in Figure 2.

In contrast to classical compartment models, we consider the whole population and their contacts in networks. Each individual in the community can be regarded as a vertex in the network, and each contact between two individuals is represented as an edge (line) connecting the vertices. The number of edges emanating from a vertex — that is, the number of contacts a person has — is called the degree of the vertex. Therefore, we assume that the population is divided into *n* distinct groups of sizes *N_k_* (*k* = 1, 2, …, *n*) such that each individual in group *k* has exactly *k* contacts per day. If the whole population size is *N* (*N* = *N*_1_*+ N*_2_ + ⋯ + *N_n_*), then the probability that a uniformly chosen individual has *k* contacts is *P*(*k*) = *N_k_*/*N*, which is called the degree distributions of the network. Empirical studies have shown that many real networks have scale-free (SF) degree distributions *P*(*k*) *≈ k^–γ^* with 2 *≤* γ *≤* 3 where the epidemic model does not show an epidemic threshold (see [18]) and Poisson degree distributions *P*(*k*) = *µ^k^*/*k*! exp(*–µ*) (see [19]). If *S_k_* , *E_k_*, *A_k_*, *I_k_* and *R_k_* represent the number of susceptible, exposed, asymptomatically infected, symptomatically infected and recovered individuals within group *k* (where *S_k_* + *E_k_* + *A_k_* + *I_k_* + *R_k_* = *N_k_*), then the following system of differential equations captures disease spread for arbitrarily large networks (*N → ∞*), for both transmission through the network and the mean-field type transmission(1)(2)(3)(4)(5)

where  represent the expectation that any given edge points to an infected and asymptomatically infected vertex respectively. Note that ; thus, *S_k_*(*t*) + *E_k_*(*t*) + *A_k_*(*t*) + *I_k_*(*t*) + *R_k_*(*t*) = *N_k_* is constant.

The densities of susceptible, exposed, asymptomatically infected, symptomatically infected and recovered nodes of degree *k* at time *t*, are denoted by *s_k_*, *e_k_*, *a_k_*, *i_k_* and *r_k_*, respectively. If *S_k_*, *E_k_*, *A_k_*, *I_k_* and *R_k_* are used to represent *s_k_*, *e_k_*, *a_k_*, *i_k_*, and *r_k_* respectively, we can still use system (1)-(5) to describe the spread of disease on the network. Clearly, these variables obey the normalization condition

*S_k_* + *E_k_* + *A_k_* + *I_k_* + *R_k_* = 1, and also

All parameters are positive constants and we summarize them in Table 1.

**Table 1:** Parameters of the model

Parameters	description
*λ*_1_	transmission coefficient between community *S_k_* and *A_i_*
*λ*_2_	transmission coefficient between community *S_k_* and *I_i_*
*δ*	rate of becoming infectious after latentcy
*γ*	rate of becoming asymptomatically infected
1* – γ*	rate of becoming symptomatically infected
*α*_1_	recovery rate of asymptomatically infected
*α*_2_	recovery rate of symptomatically infected

The mathematical formulation of the epidemic modelling on the network is completed with the initial conditions given as *S_k_*(0) = *S_k_*_0_, *I_k_*(0) = *I_k_*_0_, *E_k_*(0) = *A_k_*(0) = *R_k_*(0) = 0*.*

## Analysis

### Stability and basic reproduction number

One can verify that system (1)-(5) has a unique infection-free equilibrium *P*^0^ (1, ⋯ , 1, ⋯ ,1, 0, 0, ⋯ , 0). Following van den Driessche and Watmough [20], we note that only compartments *E_k_*, *A_k_* and *I_k_* are involved in the calculation of *R*_0_. In the infection-free state *P*^0^, the rate of appearance of new infections *F* and the rate of transfer of individuals out of the two compartments *V* are given by

where  are zero matrices,

and

Using the concepts of next-generation matrix [20], the reproduction number is given by *R*_0_ = *ρ*(*FV^–^*^1^), the spectral radius of the matrix *FV^–^*^1^.

To determine the spectral radius of *FV^–^*^1^, we first represent the inverse of *V* by the following matrix:

Setting *C* = *FV^–^*^1^, we have

where  are zero matrices and

Now we are ready to compute the eigenvalues of the matrix *C* = *FV^–^*^1^.

Obviously, *C* and  have the same spectral radius. Since matrix  has rank 1, the spectral radius  is equal to the trace of . Note that

Therefore, we obtain the reproductive number(6)

In summary, we have the following theorem.

**Theorem 1** If *R*_0_ < 1, the infection-free equilibrium *P*^0^(1, ⋯ , 1, ⋯ , 1, 0, 0, ⋯ , 0) of system (1)-(5) is locally asymptotically stable, and if *R*_0_ > 1 the infection-free equilibrium *P*^0^ is unstable.

Next, we will prove the global asymptotic stability of the infection-free equilibrium.

**Theorem 2** If *R*_0_ < 1, the infection-free equilibrium *P*^0^(1, ⋯ , 1, ⋯ , 1, 0, 0, ⋯ , 0) of system (1)-(5) is global asymptotically stable.

**Proof.** Let us consider the Lyapunov function of the form:

where .

We now compute the time derivative of *L*(*t*) along the solutions of system (1)-(5). It is seen that

Furthermore, *L′*(*t*) = 0 only if *A_k_* = *I_k_* = 0. Therefore, the global stability of *P*^0^ when *R_0_* < 1 follows from LaSalle’s Invariance Principle [21].

## Estimation of parameters

To calculate the basic reproduction number for the H1N1 epidemic in China and to explore the transient dynamics of the transmission under different vaccination schemes, we need to estimate the model parameters. In general, parameters of a model as system (1)-(5) can be estimated via least-squares fitting of the model solution to the observed data *i*(*t*) [22-24]. In other words, we are looking for the set of parameters Λ = (λ_1_, λ_2_, γ, δ, α_1_, α_2_) such that the associated model solution best fits the epidemic data by minimizing the sum of the squared differences between the observed data *i*(*t*) and the total number . Therefore, we need to minimize the objective function:

where *n_d_* represents the number of days we choose from the observed data.

In the real world, *P*(*k*) usually obeys a power-law distribution. Hence, *P*(*k*) = 2*m^2^k^–ν^* (*m* = 3 and *ν* = 3*.*5) is used in model (1)-(5).

**Figure 3:**
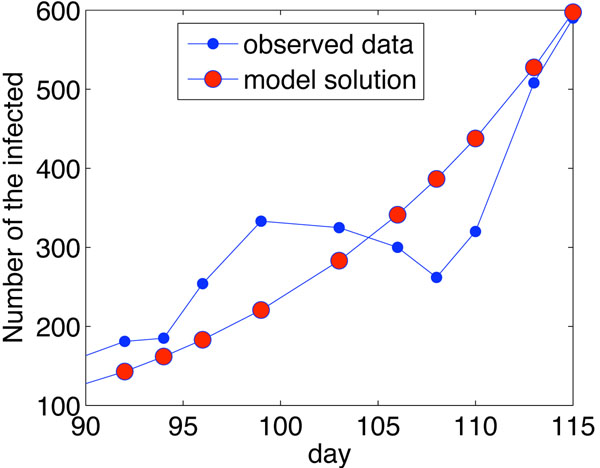
**Observed data and model simulation.** Observed data and model simulation of the number of infected individuals. The minimum degree is 3 and maximum degree is 100.

For the estimation of parameters, accurate data are essential. Due to the complexity of the spreading of H1N1 and adjustment of control strategics in China, official data are not available for our modeling studies. Luckily, for the period from August 9th to September 2nd, the reported infected data contain useful information: the number of the infected and recovered classes from the daily data. The data reported in this period are almost continuous, and there is no vaccination in this time period. Therefore, we use the data collected from August 9th to September 2nd in China to estimate the parameters of model (1)-(5). For the simulations, we set the step size as ∆*t* = 0*.*04. By using Euler and the advanced alternate directions scheme [22], we estimate the parameters and summarize them in Table 2. Using the parameters in Table 1, a straightforward computation using formula (6) gives the basic reproductive number for the H1N1 epidemic in China as *R*_0_ = 1*.*6809. Using the parameters in Table 2, we compared the model simulation results with the observation data in Figure 3. It can be seen that our model captures the main features of the spread of the H1N1 in China.

**Table 2:** Parameters estimated from the observed data in China

Parameters	Estimated value
*λ_1_*	0.01
*λ_2_*	0.188
*δ*	0.4
*γ*	0.85
*α_1_*	0.141
*α_2_*	0.141

## The effect of vaccination strategies

Vaccination is very powerful in controlling influenza. In this section, we will discuss the impact of various immunization schemes.

### Uniform immunization strategy

Uniform immunization strategy is the simplest immunization schemes [14,25,26]. Using *p* for the immunization rate (0 <*p* < 1), by substituting *λ*_1_*→* (1 – *p*)*λ*_1_ and *λ*_2_*→* (1 – *p*)*λ*_2_ in model (1)-(5), the model becomes

We obtain the critical fraction *p_c_* for the prevention and control of the prevalence of H1N1 as . For the case of China, this is . In other words, in order to control the prevalence, at least 40% of the whole susceptible population would have to be immunized through vaccination (about 536 million individuals).

### Targeted immunization

Another effective strategy is the targeted immunization [25,26]. For the network, we introduce lower and upper thresholds *κ*_1_ and *κ*_2_, such that if *k* >*κ*_2_, all nodes with connectivity *k* are immunized, while if *κ*_1_ <*k* <*κ*_2_, *p_k_* (0 <*p_k_ ≤* 1) portion will be immunized, and *p_k_* is defined as the fraction of individuals to be immunized, i.e., we define the immunization rate *σ_k_* as(7)

where  is the average immunization rate. The epidemic model (1)-(5) now becomes(8)

**Figure 4:**
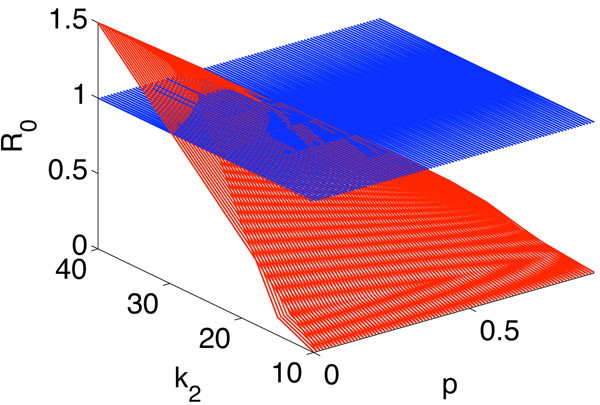
***R*_0_ as a function of *k*_2_ and *p****. R*_0_ is plotted as a function of *k*_2_ and *p.* Here, *k*_1_ = 10, *λ*_1_ = *λ*_2_ = 0*.*0104, *δ* = 0*.*4, *α*_1_ = *α*_2_ = 1/7 and *P*(*k*) = 2*m^2^k^–v^* (*m* = 3 and *ν* = 3*.*5).

We then calculate the reproductive number to obtain

or

where, for convenience, we set *p_k_* = *p* in model (7). We plot *R*_0_ as a function of *k*_2_ and *p* in Figure 4. One can see from this figure that *R*_0_ is an increasing function of *k*_2_ but a decreasing function of *p.* In other words, if *p* is large or *k*_2_ is small, more people receive vaccination, then H1N1 can be controlled.

## The final size relation

First, we show that for the model (1)-(5) the disease will eventually die out, i.e., *A*(∞) = 0, *E*(∞) = 0, and *I*(∞) = 0.

Note that the positive orthant is invariant, so all solutions of model (1)-(5) remain non-negative and bounded in the set defined by *S_k_*, *A_k_*, *I_k_*, *R_k_* ≥ 0 and *S_k_ + A_k_ + I_k_ + R_k_* = *1.* Observing that(9)

we see that *S_k_*(*t*) + *E_k_*(*t*) is decreasing whenever *E_k_* > 0. However, *S_k_* + *E_k_* is bounded below by 0; hence, it has a limit. Moreover, model (1)-(5) implies that  is bounded because *E_k_*(*t*) is bounded. Hence , so *E_k_*(*∞*) = 0. Similarly, we can prove that *A*(*∞*) = 0 and *I*(*∞*) = 0. We adopt the convention that, for an arbitrary continuous function *w*(*t*) with non-negative components, . If we integrate the seventh equation from *t* = 0 to *∞*, we have(10)

The left-hand side of (10) is finite because the components of *S_k_*(0), *S_k_*(*∞*), *E_k_*(0) and *E_k_*(*∞*) are bounded by the initial total population size. Therefore, the right-hand side of (10) is also finite and *δ* is positive. Since *E_k_*(*∞*) = 0, we have

Similarly, we can obtain

and

### The final size without vaccination

Integration of equation (1)) from 0 to *t* gives

Letting *t → ∞*, we have

If *S_k_*(0) = *S_k_*_0_, *I_k_*(0) = *I_k_*_0_, *E_k_*(0) = *A_k_*(0) = 0, then the final size relation becomes

If *S_k_*(0) = *S_k_*_0_, *I_k_*(0) = *I_k_*_0_, *E_k_*(0) = *E_k_*_0_, *A_k_*(0) = *A_k_*_0_, then the final size relation becomes

### The final size with vaccination

If vaccination follows a uniform immunization strategy, we have

**Figure 5:**
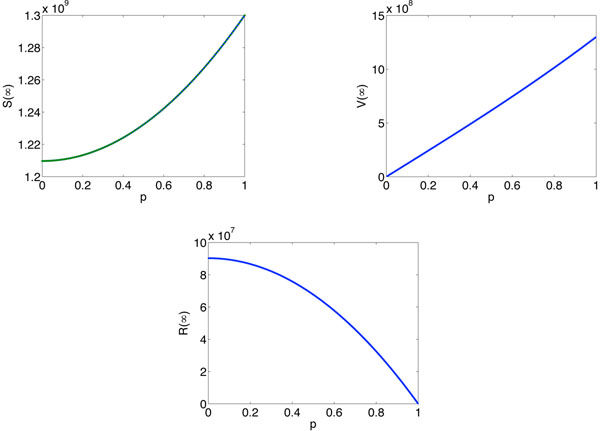
**The final sizes for susceptible, recovered and vaccinated population**. The final sizes of the susceptible, recovered and vaccinated are plotted as a function of *p.* We use parameters *λ*_1_ = 0*.*01, *λ*_2_ = 0*.*188, *δ* = 0*.*4, *α*_1_ = *α*_2_ = 0*.*141, *γ* = 0*.*85 and *P*(*k*) = 2*m*^2^*k^–ν^* (*m* = 3 and *ν* = 3*.*5).

To fully see the effect of vaccination, we show that the final size of susceptible, recovered and vaccinated individuals. It can be seen from Figure 5 that the final size of the susceptible and vaccinated increase as *p* increases. However, the final size of the recovered is a decreasing function of *p*.

If vaccination is a targeted immunization, the final size relation becomes

### The final size with vaccination from time *T*

If the vaccination strategy from time *T* follows a targeted immunization scheme, integration of equation (1) from 0 to *T* and integration of equation (8) from *T* to *t* (*t* >*T*) gives

Letting *t → ∞*, we can obtain the final size relation with targeted immunization scheme from time *T*

## Conclusions

Network models can capture the main features of the spread of the H1N1. In this paper, using a network epidemic model for influenza A (H1N1) in China, we calculated the basic reproduction number *R*_0_ and discussed the local and global dynamical behaviors of the disease-free equilibrium. The effects of various immunization schemes were studied and compared. A final size relation was derived for the network epidemic models. The derivation depends on an explicit formula for the basic reproduction number of network disease transmission models. The transmission coefficients are estimated through least-squares fitting of the model to observed data of the cumulative number of hospital notifications. We also gave the estimated value for the reproduction number for influenza A (H1N1) in China as *R*_0_ = 1*.*6809.

Parameters were estimated during the period when the vaccination was not applied. For these parameters, we found that *γ* = 0*.*85, which means that 15% of the exposed become infected during the early course of the endemic. Although vaccination commenced in China in November 2009, we were not able to compare the real data with the model projections due to lack of data.

## List of abbreviations used

H1N1: Swine Influenza A; WHO: World Health Organization; CDC: Center for Disease Control; GAS: Global Asymptotic Stability; SF: Scale-Free.

## Competing interests

The authors declare that they have no competing interests.

## Authors’ contributions

ZJ, HZ and JK designed the research and proposed the model, ZJ and JZ performed the mathematical analysis, and L-PS and G-QS ran the numerical simulations. All authors read and approved the manuscript.
